# Identification and characterization of locus-specific methylation patterns within novel loci undergoing hypermethylation during breast cancer pathogenesis

**DOI:** 10.1186/bcr3612

**Published:** 2014-02-03

**Authors:** Tomasz K Wojdacz, Johanne A Windeløv, Britta B Thestrup, Tine E Damsgaard, Jens Overgaard, Lise Lotte Hansen

**Affiliations:** 1 Department of Biomedicine, University of Aarhus, Wilhelm Meyers Allé 4, The Bartholin Building, DK-8000 Aarhus C, Denmark; 2 Department of Experimental Clinical Oncology, Aarhus University Hospital, Nørrebrogade 44, DK-8000 Aarhus C, Denmark; 3 Department of Plastic Surgery, Aarhus University Hospital, Nørrebrogade 44, DK-8000 Aarhus C, Denmark

## Abstract

**Introduction:**

Despite similar clinical and pathological features, large numbers of breast cancer patients experience different outcomes of the disease. This, together with the fact that the incidence of breast cancer is growing worldwide, emphasizes an urgent need for identification of new biomarkers for early cancer detection and stratification of patients.

**Methods:**

We used ultrahigh-resolution microarrays to compare genomewide methylation patterns of breast carcinomas (*n* = 20) and nonmalignant breast tissue (*n* = 5). Biomarker properties of a subset of discovered differentially methylated regions (DMRs) were validated using methylation-sensitive high-resolution melting (MS-HRM) in a case–control study on a panel of breast carcinomas (*n* = 275) and non-malignant controls (*n* = 74).

**Results:**

On the basis of microarray results, we selected 19 DMRs for large-scale screening of cases and controls. Analysis of the screening results showed that all DMRs tested displayed significant gains of methylation in the cancer tissue compared to the levels in control tissue. Interestingly, we observed two types of locus-specific methylation, with loci undergoing either predominantly full or heterogeneous methylation during carcinogenesis. Almost all tested DMRs (17 of 19) displayed low-level methylation in nonmalignant breast tissue, independently of locus-specific methylation patterns in cases.

**Conclusions:**

Specific loci can undergo either heterogeneous or full methylation during carcinogenesis, and loci hypermethylated in cancer frequently show low-level methylation in nonmalignant tissue.

## Introduction

Denmark has the highest standardized incidence of breast cancer in the world, with over 4,500 new cases annually [1]. The breast cancer incidence has increased since the mid-1980s, and, despite the fact that early detection combined with specialized treatment has significantly improved the survival of cancer patients, the disease still presents a problem for healthcare systems. Breast cancer treatment, as well as treatment of any other cancer, can be approached at two levels: first, early detection, which is critical for long-term survival of the patients, and second, personalized patient care, which potentially can become the most successful approach in cancer treatment. Both of these approaches require biomarkers for patient identification and stratification.

Methylation is a well-established epigenetic process of gene expression regulation. In general terms, methylation of promoter sequences of protein-coding genes results in transcriptional downregulation of the gene, and hypomethylation of previously methylated promoter regions permits transcription [2]. Two adverse phenomena characterize the process of carcinogenesis: locus-specific hypermethylation and global depletion of methyl groups from cancer genomes. Hypermethylation of promoters has been widely shown to contribute to silencing of tumor suppressor genes during carcinogenesis. Global hypomethylation of the cancer genome was initially shown to cause genomewide allelic instability, but recently the involvement of this process in transcriptional gene regulation has become increasingly recognized [2].

DNA methylation changes have been shown to take part in the very first steps of neoplastic transformation, which makes methylation biomarkers very attractive targets for early cancer detection [3]. Moreover, many phenotypical features of the cancer are a consequence of the methylation changes. Those changes are predominantly cancer type–specific and therefore have potential to be powerful biomarkers for cancer patient stratification [4].

In general, clinically useful biomarkers must be applicable in one of the clinical disease management areas: diagnostics, prognostication and treatment monitoring. More than three decades of epigenetic research have provided strong research evidence that methylation-based biomarkers can be applied in all the above areas of clinical use. Nevertheless, current use of methylation biomarkers in clinical cancer management is very limited. The difficulties in clinical implementation of the methylation biomarkers can be attributed mainly to low number of studies focused on extensive clinical validation of the novel (and known) biomarkers.

A study aiming to develop a methylation biomarker for clinical use should, apart from utilizing state-of-the-art methylation detection methodologies, consist of five steps:

1. Discovery, whereby, in most of the cases genomewide screening is applied in a search for the candidate biomarkers;

2. Initial clinical validation, whereby each candidate biomarker must be shown to provide an ability to distinguish nonmalignant healthy tissue from malignant cancer tissue;

3. Retrospective validation, whereby archival material is used to determine if there is a significant correlation between specific methylation changes and the disease phenotype. (Detailed records accompanying patient samples are critical for this part of the biomarker development process, and it is highly advisable to use samples from various patient populations in these studies.);

4. Prospective validation, whereby the biomarker is used in clinical trials; and

5. Long-term monitoring of the biomarker’s clinical use after the above-described biomarker development process to assess its impact.

Herein we present and discuss the results of the first two stages of the proposed biomarker development process for breast cancer.

## Methods

### Clinical sample material

Twenty freshly frozen breast carcinomas were obtained from Aarhus University Hospital, and DNA was extracted from those samples using the DNeasy Blood & Tissue Kit (QIAGEN, Hilden, Germany) according to the manufacturer’s protocol. Seventy-four nonmalignant breast tissue samples from breast reduction surgeries were collected at the Department of Plastic Surgery, Aarhus University Hospital. The women undergoing breast reduction surgery had had mammograms, and only women without signs of malignancies were enrolled in this study. The breast tissue obtained from breast reductions can potentially differ from healthy breast tissue; however, because of ethical considerations, this type of control material was the only available source for our experiments. DNA taken from breast reduction samples was also extracted using the DNeasy Blood & Tissue Kit. Tumor DNA for screening analyses was obtained from 274 patients diagnosed with sporadic breast cancer. The DNA samples were collected between 1992 and 1994 at Aarhus University Hospital. Complete information about the breast cancer cohort and DNA extraction procedure were previously published [5,6]. Patient consent for our use of the human material in this study was obtained and approved by the Research Ethics Committee of Mid Jutland, Denmark.

### Microarray analyses

DNA for microarray experiments was extracted from 20 freshly frozen tumor tissue samples. After extraction, the methylated DNA fragments were enriched in each sample using a methylated DNA immunoprecipitation (MeDIP) protocol. A detailed description of the procedure can be found in Additional file 1. The same procedure was applied for five tissue samples taken from women who had undergone breast reduction surgery, and these served as a control for the microarray experiments. Two fractions from each sample (MeDIP enriched and input) were labeled with Cy5 and Cy3, respectively, and cohybridized to the NimbleGen Human DNA Methylation 2.1M Deluxe Promoter Array (Roche/NimbleGen, Madison, WI, USA). Arrays were processed using NimbleScan software (Roche/NimbleGen) to produce log_2_ signal ratios at each probe. These ratios were averaged within each class of sample to produce a single set of mean ratios per class. The mean ratio sets were processed again with NimbleScan software to generate a relative enrichment score at each probe for each class using a Kolmogorov-Smirnov test in a window (750 bp) around each probe. The enrichment scores for the mean ratios of each class were subtracted to produce a differential score indicating enrichment or depletion of signal in one group relative to the other, and a significance threshold of 2 was applied to the differential scores. Two or more consecutive significant differential scores within 500 bases of each other constituted a differentially methylated region (DMR). Each DMR was mapped to the genome using NimbleScan software. Lists of the annotated regions can be found in Additional file 2. For the results presented in this paper, only the list of DMRs computed by subtraction of tumors from controls (hypermethylated DMRs) was manually mined for the candidate biomarkers. The potential candidate biomarkers (total of 24) with the highest differential scores were selected for validation analyses. All validation experiments were performed using methylation-sensitive high-resolution melting (MS-HRM) and verified by Sanger sequencing. The microarray data were deposited in the Gene Expression Omnibus database (GEO) database [GEO:GSE43095].

### Methylation-sensitive high-resolution melting

All MS-HRM assays were designed according to previously published guidelines and optimized to allow for highly sensitive methylation detection [7,8]. In each run, a range of standards was included to control for unbiased sensitivity of the detection: 0% (unmethylated: EpiTect Control DNA; QIAGEN), 1% and 10% (fully methylated template in an unmethylated background) and 100% (methylated: EpiTect Control DNA). MS-HRM amplification was performed in triplicates. PCR mix consisted of 1× LightCycler 480 High Resolution Master mix (Roche, Penzberg, Germany), 3 mM Mg^2+^, 250 to 500 nM concentrations of each primer and 6 ng of template. PCR amplifications and HRM analyses were performed on the LightCycler 480 platform (Roche) and included 50 cycles at the assay-specific parameters described in Additional file 3. The conditions and primer sequences used in MS-HRM experiments are listed in Additional file 3. Bisulfite conversions of the clinical samples were performed with the EZ DNA Methylation-Gold Kit (ZymoResearch, Irvine, CA, USA).

### Sequencing analyses

To confirm the MS-HRM results, a subset of MS-HRM PCR products was sequenced using the Sanger method as previously described [9]. In brief, PCR products obtained from MS-HRM analyses were directly sequenced using the same primers as those used for the MS-HRM analyses. To decrease the costs and labor required for the sequencing, the forward strand was sequenced from all the representative samples. In cases of ambiguous results, the reverse strand was also sequenced. Overall, we performed more than 300 sequencing reactions to confirm the MS-HRM results. Despite the fact that we attempted to sequence very small PCR products (around 100 bp; for details, see Additional file 3), we successfully and reproducibly confirmed the methylation status for each HRM profile group described herein (see Results). We were not able to sequence WT1 and SIX6 PCR products (a very short PCR product), but our high confidence in the sequencing data derived from all other assays allowed us to generalize the results for those two DMRs. The sequencing data for samples displaying low-level methylation for the HTR1B MS-HRM assay did not show methylation. However taking into account both superior sensitivity of the MS-HRM over sequencing [9] and the fact that all other low-methylation profiles showed methylation on the sequencing data, we have classified these samples as low-level methylated (Table 1).

**Table 1:** Frequencies of DNA methylation in the cancer tissue samples

**Loci ID**	**Samples available**^**a**^	**Methylation-negative, *n *** **(%)**	**Low methylation, *n *** **(%)**^**b**^	**Heterogeneous methylation, *n *** **(%)**	**Full methylation, *n *** **(%)**	**Both alleles present, *n *** **(%)**
*TITF1*	220	0 (0.0)	38 (17.3)	0	145 (65.9)	37 (16.8)
*HOXB13*	239	18 (7.5)	20 (8.4)	0	23 (9.6)	178 (74.5)
*NR2E1*	164	15 (9.1)	14 (8.5)	18 (11.0)	89 (54.3)	28 (17.1)
*HTR1B*	162	28 (17.3)	68 (42.0)	20 (12.3)	4 (2.5)	42 (25.9)
*HMX2*	187	9 (4.8)	1 (0.5)	13 (7.0)	164 (87.7)	0
*BC008699*	261	4 (1.5)	4 (1.5)	26 (10.0)	206 (78.9)	21 (8.0)
*SLC38A4*	234	0	16 (6.8)	93 (39.7)	80 (34.2)	45 (19.2)
*FLJ32447*	264	0	28 (10.6)	191 (72.3)	48 (18.2)	0
*WT1*	218	44 (20.2)	0	178 (81.7)	21 (9.6)	0
*TMEM132D*	218	6 (2.8)	21 (9.6)	154 (70.6)	16 (7.3)	21 (9.6)
*NKX2-3*	169	0	16 (9.5)	108 (63.9)	38 (22.5)	7 (4.1)
*GHSR*	260	56 (21.5)	24 (9.2)	180 (69.2)	0	0
*ONECUT*	243	0	16 (9.5)	134 (55.1)	63 (25.9)	30 (12.3)
*LHX1*	243	0	28 (11.5)	145 (59.7)	41 (16.9)	29 (11.9)
*SIX6*	246	5 (2.0)	11 (4.5)	173 (70.3)	55 (22.4)	2 (0.8)
*CA10*	230	21 (9.1)	48 (20.9)	137 (59.6)	24 (10.4)	0
*CHR*	252	0	44 (17.5)	192 (76.2)	16 (6.3)	0
*POU4F*	255	10 (3.9)	6 (2.4)	195 (76.5)	44 (17.3)	0
*PHOX2B*	256	0	3 (1.2)	199 (77.7)	53 (20.7)	1 (0.4)

^a^Variable numbers of the samples are reported because of clinical sample limitations. ^b^Low methylation is referred to as methylation similar to the methylation observed in control samples.

### Statistical analyses

The statistical analyses were performed with the Stata 10.1 software package (StataCorp, College Station, TX, USA) and R statistics.

## Results

### Identification of hypermethylated loci

In the first part of this study, we focused on identification of hypermethylated DMRs in breast cancer. NimbleScan mapping of DMRs extracted from array data showed that DMRs detected in our sample panel could potentially be associated with more than 1,000 functional genomic elements (see Additional file 2 for a complete list and coordinates of the DMRs). A direct indication of the accuracy of this part of our experimental approach was the finding that loci previously reported to undergo hypermethylation during breast cancer pathogenesis, such as for example*PAX2*, *MYOD1* and *PITX2,* were also detected in our microarray experiments. For each called DMR, our microarray data analysis workflow (see Methods) provided us with a differential score. This value is in principle a derivative of the methylation difference between cases and controls. We observed that the higher the score, the more pronounced the methylation gain, when we compared both cases and controls. Therefore, 23 DMRs with the highest differential score were selected for the MS-HRM-based microarray validation experiments. MS-HRM assays were targeted to the called DMR or to the closest region that potentially could undergo differential methylation (for example, a CpG island (CGI)). The results of the MS-HRM microarray validation corroborated the microarray results for 21 of the selected target sequences (unpublished data [7]). At this point in our experimental procedure, the MS-HRM results indicated low-level methylation in the control samples for a subset of the assays. However, the methylation level of those loci was significantly higher in cases. The use of quantitative properties of MS-HRM in this experiment was therefore critical [7,10]. At the same time, this finding indicates that the microarray used in our experiments is able to robustly detect small relative differences in methylation between cases and controls.

Two of the MS-HRM assays used in the microarray validation experiments did not confirm the microarray results. One possible explanation is that PCR-based MS-HRM assays cover only approximately 100 bp, whereas the region with aberrant methylation called on the microarray can span large genomic regions and the methylation status within those sequence can differ [11]. Design of PCR assays without prior knowledge of the methylation changes throughout the region is still challenging and can simply result in targeting the part of the regions that does not undergo cancer-dependent methylation changes. This explanation does not rule out the possible technological limitations of the microarray technology, however, which can lead to false discoveries. Despite the fact that false-positive results were present at a very low rate in our data set, the fact that they were present at all underlines the critical importance of validation of the results obtained by any genomewide screening technology by using PCR-based methods.

### Initial clinical validation

All DMRs positively validated in the microarray validation experiments were subjected to initial clinical validation screening. In principle, this part of the biomarker development workflow aims to show (with a statistically sound sample number) the potential of the discovered DMR to distinguish between cancer (cases) and healthy controls. We have screened 275 samples of breast cancer with the same MS-HRM assays used in the microarray validation process as well as 74 DNA samples from breast reduction surgeries (representing healthy controls). The overall results of the initial clinical validation screening for the 19 DMRs are presented in Tables 1 and 2. The results of the screening for three of the DMRs are not included in the tables because the MS-HRM data for those DMRs were challenging to interpret. Further analyses of those loci have to be performed to clarify whether those DMRs are affected by specific genetic events occurring during breast tumor carcinogenesis (data not shown). The samples from the control group were subclassified into three groups on the basis of the MS-HRM results (Table 2): (1) samples displaying no methylation; (2) samples showing low levels of methylation with *low-level methylation* defined as methylation less than 1% standard or any aberrations from the unmethylated profile; and (3) methylation-positive samples. Interestingly, the cancer samples displayed a significant variety of the HRM profiles. The MS-HRM technology allowed us to evaluate heterogeneous methylation in each sample screened. Heterogeneous methylation is referred to as the presence in the sample of multiple epialleles, each with a different pattern of methylated and unmethylated CpG sites for a given region. Heterogeneously methylated samples display a characteristic HRM profile for the complex mixture of heteroduplexes formed between strands that only differ at a few CpG sites. The evaluation of heterogeneous methylation was not previously performed on a large scale owing to technological limitations of the methods for methylation screening.

**Table 2:** Frequencies of DNA methylation in control tissue samples

**Loci ID**	**Samples**^**a**^**, *n***	**Low methylation**^**b**^**, *n *** **(%)**	**Methylation-negative, *n *** **(%)**	**Methylation-positive**^**c**^**, *n *** **(%)**
*TITF1*	72	47 (65.3)	25 (34.7)	0
*HOXB13*	72	13 (18.1)	59 (81.9)	0
*NR2E1*	72	6 (8.3)	66 (91.7)	0
*HTR1B*	69	0	69 (100.0)	0
*HMX2*	69	45^d^ (65.2)	24 (34.8)	0
*BC008699*	72	21 (29.2)	48 (66.7)	3 (4.2)
*SLC38A4*	62	30 (48.4)	29 (46.8)	3 (4.8)
*FLJ32447*	70	48 (68.6)	22 (31.4)	0
*WT1*	72	2 (2.8)	70 (97.2)	0
*TMEM132D*	70	37 (52.9)	33 (47.1)	0
*NKX2-3*	68	68 (100.0)	0	0
*GHSR*	72	2 (2.8)	70 (97.2)	0
*ONECUT*	71	24 (33.8)	47 (66.2)	0
*LHX1*	72	0	54 (75.0)	18 (25.0)
*SIX6*	69	8 (11.6)	61 (88.4)	0
*CA10*	47	24 (51.1)	23 (48.9)	0
*CHR*	72	72 (100.0)	0	0
*POU4F*	37	11 (29.7)	26 (70.3)	0
*PHOX2B*	72	72 (100.0)	0	0

^a^Variable number of samples is reported due to clinical sample limitations. ^b^Low methylation is referred to as methylation similar to the methylation level observed in the 1% methylation standard. ^c^Methylation-positive samples are referred to as samples displaying a HRM profile characteristic for cases. ^d^Methylation level similar to the one observed in the 1% to 10% methylation standard.

**Figure 1:**
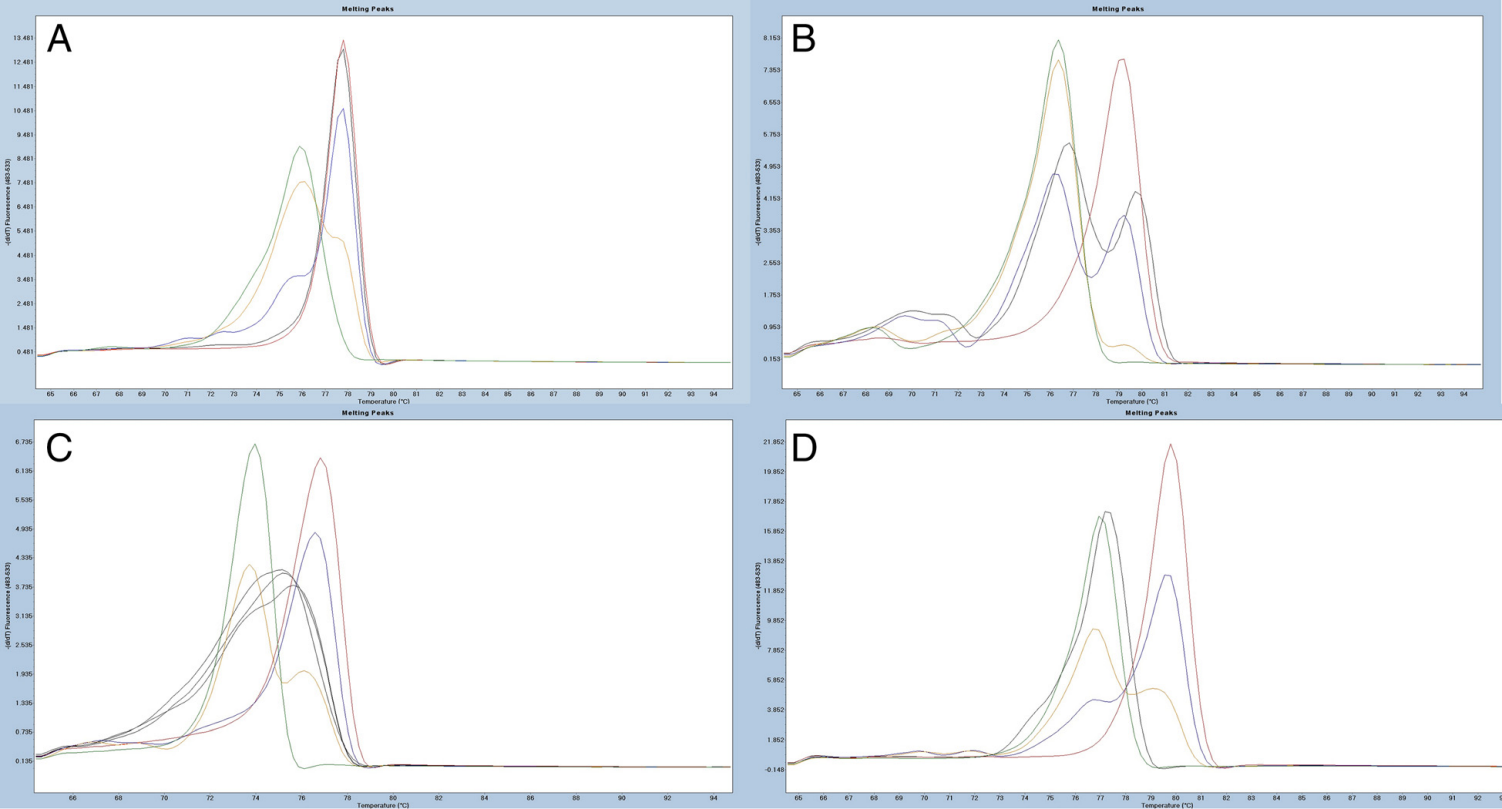
**Examples of the classes of methylation-sensitive high-resolution melting profiles observed in the case sample material.** Standards: 100% methylated (red), 10% methylated in a background of unmethylated template (blue), 1% methylated (yellow) and unmethylated (green). Sample with a representative methylation-sensitive high-resolution melting profile is shown in black. Melting peaks were generated by taking the negative derivative (d) of the melting curve data divided by the derivative with respect to time -(d/dT). **(A)** Fully methylated sample (BC008699 assay) **(B)** Sample displaying the presence of both methylated and unmethylated alleles (HOXB13 assay). **(C)** Heterogeneously methylated sample (SIX6 assay). **(D)** Sample with no signs of methylation (CA10 assay).

The significance of heterogeneous methylation is still debated, but this phenomenon may potentially be critical for the clinical application of methylation biomarkers (see Discussion for more details). Therefore, we have chosen to subdivide our results into five groups (see Table 1): (1) methylation-positive samples, (2) methylation-negative samples, (3) samples displaying heterogeneous methylation pattern, samples showing only fully methylated melting profiles and (5) samples with both methylated and unmethylated alleles present. Figure 1 illustrates examples of the different classes of HRM profiles detected in our analyses. To confirm the accuracy of the classification of HRM profiles, we performed sequencing of a subset of the samples from each of the HRM profile groups and for each of the DMRs.

### Specificity of the biomarkers and low-level methylation in controls

**Figure 2:**
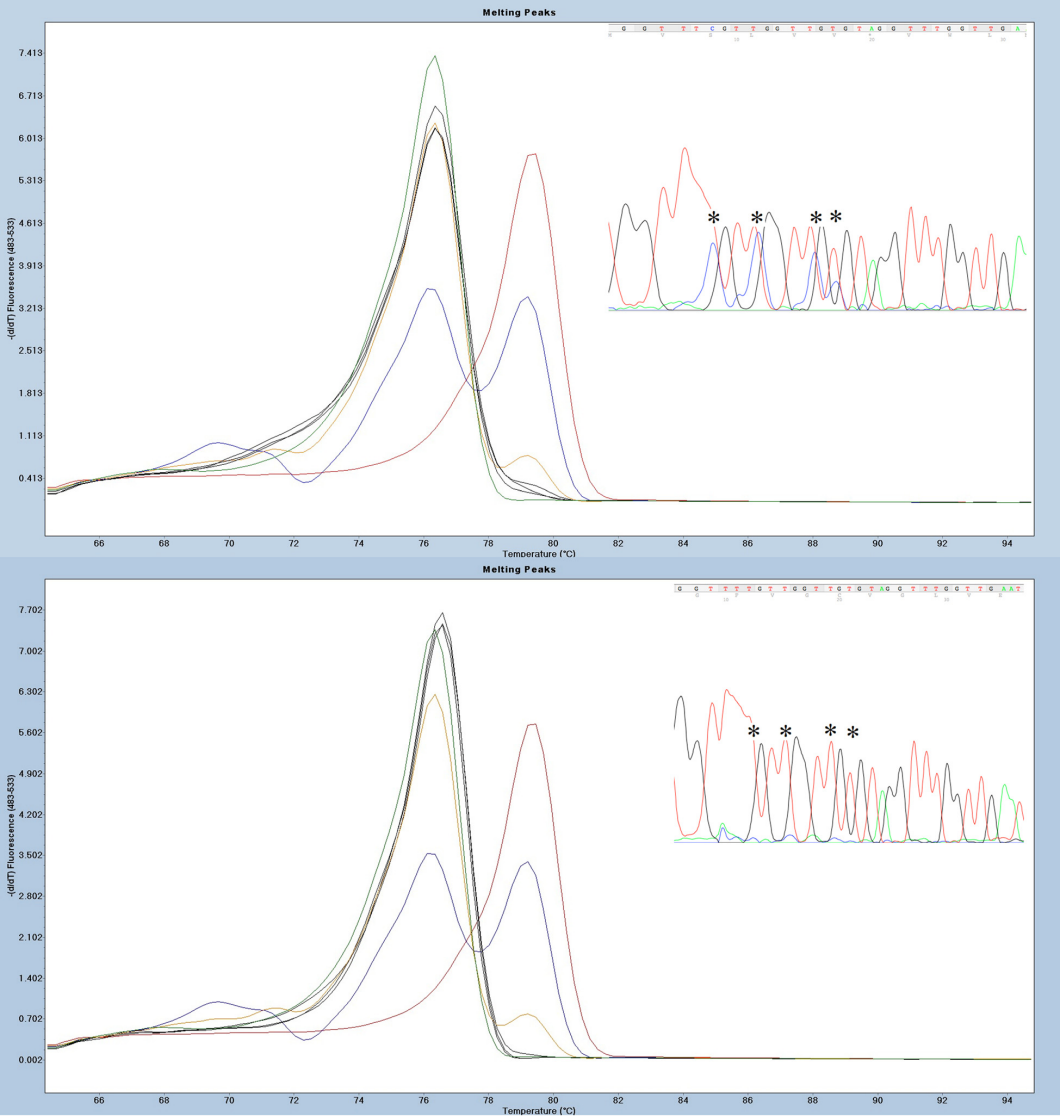
**Example of low-level methylation sample at *HOXB13 *differentially methylated region.** Standards: 100% methylated (red), 10% methylated in a background of unmethylated template (blue), 1% methylated (yellow) and unmethylated standard (green). Sample with a representative high-resolution melting (HRM) profile (black). The top panel illustrates how even small aberrations of the HRM profile from the unmethylated standard represent low methylation levels. Methylation-sensitive HRM (MS-HRM) results are shown with the sample in black and confirmation of the HRM results with sequencing where asterisks indicate double-sequences at CpG sites (both alleles present: T (unmethylated allele) and C (methylated allele)). Lower panel displays the same data for an unmethylated sample. Melting peaks were generated by taking the negative derivative (d) of the melting curve data divided by the derivative with respect to time -(d/dT).

The specificity of each of DMR was evaluated on the basis of MS-HRM screening of the control tissue. The results are presented in Table 2. Interestingly, we observed low-level methylation in control samples for 17 DMRs. The frequency of low-level methylation was as high as 100% for three of the loci (*NKX2-3*, *CHR* and *PHOX2B*). In addition, three loci (*BC008699, SLC38A4* and *LHX1*) showed low frequencies of the methylation levels, similar to those observed in cancer tissue. These loci will not be further considered for biomarker development, as their specificity most likely will be very low. Figure 2 illustrates examples of the MS-HRM scans with low methylation levels and verification of the results by sequencing. Overall, on the basis of the sequencing results, we conclude that any aberration of the HRM profile from the unmethylated standard indicates the presence of methylation in the analyzed sample. Only one DMR in our panel (*HMX2*) showed methylation levels between 1% and 10% in the control tissue samples. The methylation levels in controls at all other DMRs were always below 1% when we analyzed the data against the 1% methylation level standard. The high frequency of low levels of methylation in the control tissue hampers the specificity of the biomarkers. However, the quantitative aspect of the MS-HRM technology allows establishment of a cutoff point for low-level methylation.

### High methylation levels in cancer samples

**Figure 3:**
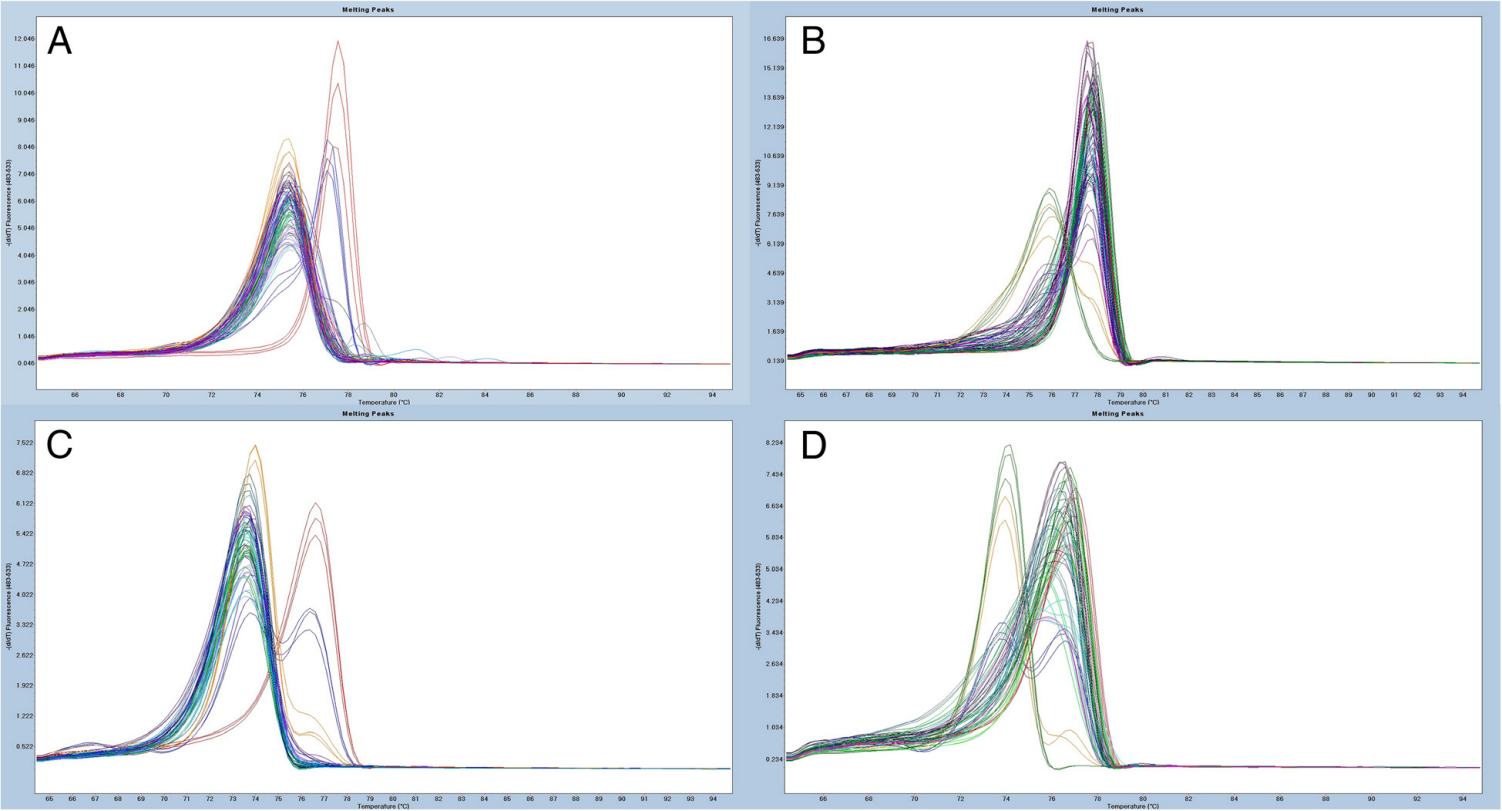
**Examples of the overall methylation screening results in cases and controls illustrating a shift in methylation of the locus during carcinogenesis.** Standards: 100% methylated (red), 10% methylated in a background of unmethylated template (blue), 1% methylated (yellow) and unmethylated standard (green). Melting peaks were generated by taking the negative derivative (d) of the melting curve data divided by the derivative with respect to time -(d/dT). Each panel displays 20 methylation-sensitive high-resolution melting scans. **(A)** and **(B)***BC008699* assay. **(C)** and **(D)***SIX6* assay. Scans from control samples are shown in **(A)** and **(C)**. **(B)** and **(D)** show HRM scans of the cancer tissue samples.

Despite the fact that 17 of the DMRs from our panel showed low levels of methylation in the control tissue, those levels seem to be insignificant compared to the levels of the methylation observed at the same locus in the cancer samples. All tested DMRs showed drastic gains of methylation during carcinogenesis. A general switch in methylation pattern from unmethylated in controls to methylated in cancer samples for two of the screened loci (*SIX6* and *BC008699*) is illustrated in Figure 3. Very few cancer samples in our cohort displayed low methylation levels similar to those observed in the control tissue (see Table 1 for details). For specificity calculations, methylation in those samples can be interpreted as a normal methylation level when a cutoff point has been established based on analyses of methylation in the control group. The cutoff points for low levels of methylation can provide 100% specificity for the methylation biomarkers. However, before a cutoff point can be established, the pathological significance of low levels of methylation within each DMR has to be evaluated.

### Two types of locus-specific methylation

The frequencies of methylation in cancer tissue samples are listed in Table 1. As shown, 12 DMRs in our panel displayed predominant gains of heterogeneous methylation affecting from 55% to 81% of the samples. Five of the DMRs showed very low frequencies of heterogeneous methylation (for example, *TITF1* and *HMX2* with 65% and 87% of the cancer samples, respectively, showing the presence of full methylation of both alleles). No heterogeneous methylation was seen for the *HOXB13* assay, but 74.5% of samples contained both methylated and unmethylated alleles at this DMR. Only one of the DMRs screened (*SLC38A4*) showed balanced frequencies of heterogeneous and full methylation of 39% and 34%, respectively.

Functional and clinical effects of hypermethylation of the DMRs from our panel are outside the scope of this paper and will be addressed in future projects. However, it is interesting that the CGI we have targeted with our *HOXB13* assay has previously been shown to undergo hypermethylation in colon cancer [12]. Aberrant expression and methylation-dependent expression of the *HOXB13* gene has been shown in cancer [13,14]. However, mutations within the *HOXB13* gene and the linkage analyses of the neighboring 17q21–22 region found this region to be involved in the development of different cancers [15,16]. Our findings support those observations, and the fact that our results seem to indicate deactivation of only one allele by DNA methylation makes this phenomenon even more intriguing.

Overall, our methylation screening results clearly show that a locus can undergo two types of methylation during carcinogenesis: either heterogeneous or full methylation (at one or both alleles). The type of aberrant methylation seems to be locus-specific, and the mechanism of this process is unknown. To the best of our knowledge, our present study is the first to show this phenomenon, and the physiological and clinical consequences of this process have to be further researched. At the same time, the high locus specificity of the observed methylation changes indicates that our results are not a technological artefact of the methodology used in this study, but instead reflect a biological phenomenon.

## Discussion

There is a strong base of research evidence to support the utility of methylation biomarkers in the entire process of clinical disease management, from screening for predisposition through detection of the condition to personalized treatment of the disease.

As mentioned in the Introduction, the process of biomarker development for clinical applications can be described in five steps. The data presented herein address the challenges of the first two steps of methylation biomarker development for clinical use: discovery and initial clinical validation. In the biomarker discovery step, microarray technologies and next-generation sequencing) are indispensable tools affording researchers, in a single experiment, the ability to uncover a landscape of methylation changes throughout a cell genome. Currently, however, the complexity of those technologies does not allow for straightforward interpretation of the genomewide screening results. The validation step is especially important when complicated statistical modeling is used to process microarray data. In the present study, we used a very simple statistical model for microarray data processing (see Methods). Despite the simple approach involving very little data processing, validation experiments were necessary. They confirmed low (but present) false discovery results from the genomewide screening. This exemplifies the importance of validation of any genomewide methylation-based study before any conclusions are drawn. At the same time, our results illustrate that simple statistical models can be very effective in the discovery of disease-dependent methylation aberrations.

We have implemented an initial clinical validation of the biomarkers as a second step in the biomarker development workflow. This step, in which we initially aimed to answer only the question whether a potential biomarker can distinguish healthy tissue from cancer tissue, allowed us to address two important, recently emerging questions in the methylation biomarker field: (1) the question of the prevalence of low-level methylation of potential biomarkers in healthy tissue and the consequences of this phenomenon for biomarker specificity and (2) the question of the significance of the evaluation of heterogeneous methylation of potential biomarkers.

The significance of interindividual differences in low-level methylation of specific loci is frequently discussed in the field of methylation biomarkers [17,18]. There is no consensus with regard to the origin of this phenomenon and its pathological significance; however, the potential involvement of this type of methylation in disease predisposition has already been shown [19]. This phenomenon has direct influence on the specificity of the methylation biomarkers because, from the biomarker development perspective, methylation in healthy tissue should not be present in order for the biomarker to be highly informative. Our data show that low-level methylation is very frequently present in healthy tissue, and our sequencing experiments provide evidence that the low-level methylation we observed in our controls is not a technological artefact. The quantitative properties of the MS-HRM technology allow definition of a cut-off point for the low levels of methylation; however, before that can be done, the pathological insignificance of the low levels methylation has to be shown for each biomarker. The high prevalence of low-level methylation demonstrated in this study underlines the notion that evaluation of the low-level methylation in healthy tissue is critical for biomarker development and that only methods that allow for a definition of a cutoff for background methylation levels are most likely to be applicable in clinical methylation biomarker testing.

As mentioned above, a second very interesting finding of our initial clinical biomarker validation experiments in a relatively large-scale study is that the frequency of heterogeneous methylation can be very high, with some loci undergoing only heterogeneous methylation during carcinogenesis. Heterogeneous methylation was previously shown to occur at some loci, but this phenomenon has never been studied extensively in a large sample. One reason could be the technological limitations of some of the methodologies used for DNA methylation assessment [9,20,21]. The MS-HRM technology allowed us to perform methylation screening with the capability of evaluating heterogeneous methylation in a large sample panel [9]. To the best of our knowledge, we show for the first time a trend for loci to undergo two types of methylation during carcinogenesis, with some loci undergoing full methylation and others heterogeneous methylation. Moreover, our results show that heterogeneous methylation seems to be as specific to the locus as full methylation. Full methylation of the locus normally abolishes transcription. Heterogeneous methylation may not be sufficient to abolish transcription of the gene, but may interfere with only the transcription process or may be a “passenger” of the carcinogenesis process. Nevertheless, these comments are only speculative, and the physiological and clinical significance of heterogeneous methylation has to be further researched in the future.

Overall, the initial clinical validation step in our biomarker development procedure has allowed us to answer two critical questions for the potential applicability of the biomarker in clinical practice; hence, in our opinion, this step is essential for fast streamlining of the biomarker validation process.

## Conclusions

Our results show that loci undergoing hypermethylation during carcinogenesis can acquire either heterogeneous or full methylation. Furthermore, low-level methylation at those loci in nonmalignant tissue can be a common event. Consequently, researchers in studies addressing the clinical applicability of hypermethylated loci as biomarkers should evaluate the clinical relevance of both specific types of methylation, as well as low-level methylation of those loci in nonmalignant tissue.

## Competing interests

TKW and LLH are listed as inventors on a patent pending application for the aspects of MS-HRM technology. TKW, JO and LLH are also listed on a patent pending application for the aspects of the biomarkers discovered during this project. Roche Diagnostic, Penzberg, Germany, has partly supported this project based on the research agreement with the University of Aarhus, Denmark (under Danish legal regulations) but had no influence on the reporting of the results. A provisional patent application has been filed for the aspects of the new biomarkers by the University of Aarhus with TKW, JO and LLH listed as inventors.

## Authors’ contributions

TKW, JAW and BBT performed the experiments and analyzed the data. TKW and LLH drafted the manuscript. TED supervised obtaining ethical approval and collecting the specimens for the project. JO and LLH supervised the project. TKW, LLH, JO and TED conceived the project. All authors contributed to the editing of the final version of the manuscript and approved final version for publication.

## Supplementary Material

Additional file 1 Detailed methylated DNA immunoprecipitation (MeDIP) protocol used in the study. Click here for file

Additional file 2 **List of the differentially methylated regions obtained after processing of microarray data as described in the Methods section.** Click here for file

Additional file 3 Details of the methylation-sensitive high-resolution melting assays used in the validation experiments. Click here for file

## References

[B1] Danish Health and Medicine Cancer Incidence in Denmark 2001 2006 Copenhagen: National Board of Health Statistics http://sundhedsstyrelsen.dk/publ/Publ2006/SESS/Cancer_incidens/Cancer_incidens_01.pdf (accessed 10 February 2014)

[B2] Stefanska B Huang J Bhattacharyya B Suderman M Hallett M Han ZG Szyf M Definition of the landscape of promoter DNA hypomethylation in liver cancer Cancer Res 2011 71 5891 5903 10.1158/0008-5472.CAN-10-3823 21747116

[B3] Suijkerbuijk KP van Diest PJ van der Wall E Improving early breast cancer detection: focus on methylation Ann Oncol 2011 22 24 29 10.1093/annonc/mdq305 20591821

[B4] Mikeska T Bock C Do H Dobrovic A DNA methylation biomarkers in cancer: progress towards clinical implementation Expert Rev Mol Diagn 2012 12 473 487 10.1586/erm.12.45 22702364

[B5] Hansen LL Andersen J Overgaard J Kruse TA Molecular genetic analysis of easily accessible breast tumour DNA, purified from tissue left over from hormone receptor measurement APMIS 1998 106 371 377 10.1111/j.1699-0463.1998.tb01359.x 9548425

[B6] Hansen LL Yilmaz M Overgaard J Andersen J Kruse TA Allelic loss of 16q23.2-24.2 is an independent marker of good prognosis in primary breast cancer Cancer Res 1998 58 2166 2169 9605761

[B7] Wojdacz TK Dobrovic A Hansen LL Methylation-sensitive high-resolution melting Nat Protoc 2008 3 1903 1908 10.1038/nprot.2008.191 19180074

[B8] Wojdacz TK Hansen LL Dobrovic A A new approach to primer design for the control of PCR bias in methylation studies BMC Res Notes 2008 1 54 10.1186/1756-0500-1-54 18710507 PMC2525644

[B9] Wojdacz TK Møller TH Thestrup BB Kristensen LS Hansen LL Limitations and advantages of MS-HRM and bisulfite sequencing for single locus methylation studies Expert Rev Mol Diagn 2010 10 575 580 10.1586/erm.10.46 20629507

[B10] Migheli F Stoccoro A Coppedè F Omar WAW Failli A Consolini R Seccia M Spisni R Miccoli P Mathers JC Migliore L Comparison study of MS-HRM and pyrosequencing techniques for quantification of *APC* and *CDKN2A* gene methylation PLoS One 2013 8 e52501 10.1371/journal.pone.0052501 23326336 PMC3543439

[B11] Everhard S Tost J El Abdalaoui H Crinière E Busato F Marie Y Gut IG Sanson M Mokhtari K Laigle-Donadey F Hoang-Xuan K Delattre JY Thillet J Identification of regions correlating MGMT promoter methylation and gene expression in glioblastomas Neuro Oncol 2009 11 348 356 10.1215/15228517-2009-001 19224763 PMC2743215

[B12] Ghoshal K Motiwala T Claus R Yan P Kutay H Datta J Majumder S Bai S Majumder A Huang T Plass C Jacob ST HOXB13, a target of DNMT3B, is methylated at an upstream CpG island, and functions as a tumor suppressor in primary colorectal tumors PLoS One 2010 5 e10338 10.1371/journal.pone.0010338 20454457 PMC2861599

[B13] Rodriguez BAT Cheng ASL Yan PS Potter D Agosto-Perez FJ Shapiro CL Huang THM Epigenetic repression of the estrogen-regulated *Homeobox B13* gene in breast cancer Carcinogenesis 2008 29 1459 1465 10.1093/carcin/bgn115 18499701 PMC2899848

[B14] Svingen T Tonissen KF Altered HOX gene expression in human skin and breast cancer cells Cancer Biol Ther 2003 2 518 523 14614318 10.4161/cbt.2.5.441

[B15] Alanee S Couch F Offit K Association of a HOXB13 variant with breast cancer N Engl J Med 2012 367 480 481 10.1056/NEJMc1205138 22853031 PMC3926433

[B16] Ewing CM Ray AM Lange EM Zuhlke KA Robbins CM Tembe WD Wiley KE Isaacs SD Johng D Wang Y Germline mutations in HOXB13 and prostate-cancer risk N Engl J Med 2012 366 141 149 10.1056/NEJMoa1110000 22236224 PMC3779870

[B17] Dobrovic A Kristensen LS DNA methylation, epimutations and cancer predisposition Int J Biochem Cell Biol 2009 41 34 39 10.1016/j.biocel.2008.09.006 18835361

[B18] Kristensen LS Raynor MP Candiloro I Dobrovic A Methylation profiling of normal individuals reveals mosaic promoter methylation of cancer-associated genes Oncotarget 2012 3 450 461 22570110 10.18632/oncotarget.480 PMC3380579

[B19] Wong EM Southey MC Fox SB Brown MA Dowty JG Jenkins MA Giles GG Hopper JL Dobrovic A Constitutional methylation of the BRCA1 promoter is specifically associated with BRCA1 mutation-associated pathology in early-onset breast cancer Cancer Prev Res (Phila) 2011 4 23 33 10.1158/1940-6207.CAPR-10-0212 20978112 PMC4030007

[B20] Candiloro IL Mikeska T Dobrovic A Assessing combined methylation-sensitive high resolution melting and pyrosequencing for the analysis of heterogeneous DNA methylation Epigenetics 2011 6 500 507 10.4161/epi.6.4.14853 21364322 PMC3100767

[B21] Mikeska T Candiloro IL Dobrovic A The implications of heterogeneous DNA methylation for the accurate quantification of methylation Epigenomics 2010 2 561 573 10.2217/epi.10.32 22121974

