# Supplementary material for: Identification and characterization of locus-specific methylation patterns within novel loci undergoing hypermethylation during breast cancer pathogenesis
Source: Breast Cancer Res. 2014 Feb 3;16(1):R17. doi: 10.1186/bcr3612 (PMC3978461; doi:10.1186/bcr3612)
Supplement: Additional file 1 — Detailed methylated DNA immunoprecipitation (MeDIP) protocol used in the study. [file bcr3612-S1.doc]

**1. MeDIP**

MeDIP was performed on the 25 samples following a specific protocol from NimbleGen, Roche.

1. DNA extraction was performed as described in DNA extraction.

2. DNA fragmentation was performed using *Mse I* restriction enzyme (5’-T↓TTA) (New England Biolabs, R0525S).

a. 6 μg of DNA from each sample was digested with 24 U of *Mse I* (10,000U/ml) overnight at 37°C in a solution containing 10Χ NEB4 buffer (provided with the *Mse I*), BSA (1μg/μl) (Invitrogen, 15561-020), and water. The reactions was stopped by heating the samples for 20 min at 65°C.

b. Samples were purified using QIAquick PCR Purification Kit (Qiagen, 28104) as described in “4. Purification”.

c. DNA concentration was measured using a NanoDrop (Thermo Scientific) and fragmentation was verified on a 2% agarose gel, using 300 ng of *Mse I* digested DNA. Fragments were in the range of 200-1,000 bp to obtain efficient immunoprecipitation.

3. Immunoprecipitation of methylated DNA.

Monoclonal mouse anti 5-methyl cytidine antibodies, 100μg/100μl (Eurogentec, I-MECY-0100) was used in a 1:1 ratio to DNA.

a. 1.25 μg of *Mse I* digested DNA was diluted to a final volume of 300 μl in TE buffer (TE buffer: 10mM TrisHCl, pH7.5, and 1mM EDTA).

b. The samples were denatured at 95°C for 10 min and immediately cooled on ice for 5 min to obtain single stranded DNA, necessary for antibody binding. Samples were kept at 4°C.

c. Control (input) DNA: 250 ng DNA, equivalent to 60 μl, was removed from each sample and stored at -20°C.

d. Immunoprecipitated (IP) DNA: 60 μl of 5X IP buffer was added to the remaining 240 μl DNA solution (5X IP buffer: 50 ml 100mM Na-phosphate (pH 7.0), 14 ml 5M NaCl, 2.5 ml 10% triton X-100 (Sigma-Adrich, 93426), and 33.5 ml water).

e. 1,3 μg antibody was added to each sample and the DNA-antibody mixture was incubated overnight at a rotating platform at 4°C.

4. Binding of DNA:Antibody mixture to beads.

a. Protein A agarose beads (Invitrogen 15918-014) was washed twice using PBS-BSA 0.1% (10X PBS: Invitrogen, 70013-032).

1. The beads were re-suspended by shaking. 48 μl of beads was added to 1.5 ml microcentrifuge tube and centrifuged at 6,000 rpm for 2 min at 4°C. Supernatant was removed.

2. 600 μl of PBS-BSA 0.1% was added to each sample and samples incubated on a rotating platform for 5 min at 4°C. Subsequently the samples were centrifuged at 6,000 rpm for 2 min at 4°C. The supernatant was removed and this step was repeated.

b. Beads were re-suspended in 24 μl 1X IP buffer (1X IP buffer: 5x diluted 5X IP buffer) and added to the DNA:Antibody mixture and incubated on a rotating platform for 2 hours at 4°C.

c. DNA:Antibody:Beads mixture was washed three times using 1X IP buffer to remove unbound unmethylated DNA from the solution. For each wash, 1 ml of 1X IP was added to the mixture and incubated on a rotating platform for 5 min at 4°C and centrifuged at 6,000 rpm for 5 min at 4°C followed by removal of supernatant.

5. Degradation of beads and antibodies.

a. Each mixture was re-suspended in 250 μl digestion buffer (5 ml 1M TrisHCl (pH 8.0), 2 ml 0.5M EDTA, 5 ml 10% SDS (Sigma- Aldrich, L-4522), and 88 ml water).

b. 7 μl of Proteinase K mix (10mg/ml) (Roche Applied Science, 03115836001) was added to the mixture to digest the beads and antibodies. The mixtures incubated overnight at a rotating platform at 55°C.

6. Purification of methylated DNA.

a. 250 μl phenol (Sigma-Aldrich, P-4557) was added to each sample. Samples were vortexed for 30 seconds and centrifuged at 14,000 rpm for 5 min at room temperature. Supernatant was transferred to a new 1,5 ml microcentrifuge tube.

b. 250 μl Chloroform:isoamyl alcohol (24:1) (Sigma-Aldrich, C0549) was added to each sample and proceeded as above.

c. 1 μl glycogen (20mg/ml) (Roche Applied Science, 10901393001) was added, followed by the addition of 20 μl 5M NaCl and 500 μl absolute ethanol (Sigma-Aldrich, E702-3).

d. The DNA was precipitated at -80°C for 30 min followed by centrifugation at 14,000 rpm for 15 min at 4°C. The supernatant was removed and discarded.

e. The pellet was washed with 500 μl 70% ice-cold ethanol (diluted absolute ethanol, (Sigma-Aldrich, E702-3) and centrifuged at 14,000 rpm for 5 min at 4°C. The supernatant was removed and the pellet was completely dried in a SpeedVac.

f. The samples were resuspended in 30 μl 10mM TrisHCl (pH 8.5) and the DNA concentration was measured using a NanoDrop. The expected DNA yield in each sample was 10-15 ng/μl.

7. Amplification of immunopecipitated (IP) and control (Input) DNA using Whole Genome Amplification Kit 2 (WGA2, Sigma-Aldrich, WGA2-50RXN)) as described in “3. WGA 2 amplification” to get higher DNA yield.

8. After each round of amplification, samples were purified using QIAquick PCR Purification Kit, Qiagen, see section “4. Purification”, and DNA concentration was measured using a NanoDrop.

**2. WGA 2 amplification**

10 ng of IP and Input DNA were used for amplification. A positive control DNA sample, Control Human Genomic DNA, is provided in the WGA2 kit (Sigma-Aldrich, WGA2-50RXN) and is also amplified using the same procedure.

1. Fragmentation.

a. 1 μl of 10x fragmentation buffer was added to each 10 μl DNA (1ng/μl) sample (IP, input, and positive control DNA sample) in a 200 μl PCR tube. The Control Human Genomic DNA (5 ng/μl) was diluted to yield 1ng/μl.

b. The solution was heated at 95°C for 4 min in a thermal cycler and subsequently cooled on ice.

2. Library preparation.

a. 2 μl of 1x Library Preparation Buffer and 1 μl Library Stabilization Solution was added to each sample. Samples were vortexed thoroughly, centrifuged briefly, heated in a thermal cycler at 95°C for 2 min, and cooled on ice.

b. 1 μl of Library Preparation Enzyme was added to each sample. Samples were vortexed thoroughly, centrifuged briefly, and run in a thermal cycler using the following program (table 1). Time in seconds or minutes?

| **Temperature** | **Time** |
| --- | --- |
| 16 | 20 |
| 24 | 20 |
| 37 | 20 |
| 75 | 5 |
| 4 | Hold |

3. Amplification.

| Table 1 Incubation program for WGA2 libary preparation. Table legends should be placed on the top of the table not the bottom |
| --- |

A master mix containing 7.5 μl 10x Amplification Master Mix, 47.5 μl Nuclease-Free water, and 5 μl WGA DNA Polymerase was added to each sample. Samples were vortexed thoroughly, centrifuged briefly, and run in a thermal cycler using the following program (table 2).

| **Step** | **Temp.** | **Time** |
| --- | --- | --- |
| Denaturation | 95 | 3 |
| 14 cycles as follows |  |  |
| Denature | 94 | 15 |
| Anneal/Extend | 65 | 5 |

Amplification of DNA was verified on a 2% agarose gel and the DNA amount was measured using a NanoDrop.

| Table 2 Amplification program for WGA2 |
| --- |

**3. WGA3 Re-amplification**

Re-amplification was performed using WGA3 (Sigma-Aldrich WGA3-50RXN).

1. 10 μl of 1 ng/μl WGA2 amplified, and purified DNA was added to a 200 μl PCR tube.

2. A amplification mix containing 7.5 μl 10x Amplification Master Mix, 47.5 μl Nuclease-Free water, 5 μl WGA DNA Polymerase, and 3 μl 10mM dNTP mix was added to each sample. Samples were vortexed thoroughly, centrifuged briefly, and run in a thermal cycler using the same program as for WGA2 amplification (table 2).

Amplification of DNA sequences was verified on a 2% agarose gel and the DNA amount was measured using a NanoDrop.

**4. Purification**

DNA samples were purified using the QIAquick PCR Purification Kit (Qiagen, 28104).

All centrifugations were performed at 13,000 rpm at room temperature.

1. 5 volumes of Buffer PB were added to 1 volume of sample and each solution was transferred to a QIAquick spin column with a collection tube. Samples were centrifuged for 1 min. Flow-through was discarded.

2. 0,75 ml of Buffer PE was added to each sample. Samples were centrifuged for 1 min and flow-through was discarded. Samples were centrifuged for 1 min again to remove ethanol in Buffer PE.

3. The spin column was transferred to a new 1,5 ml microcentrifuge tube and 30 μl of Buffer EB was placed on the QIAquick membrane. Samples stood for 1 min. before they were centrifuged for 1 min.. This step was repeated to get a higher DNA yield.

4. All samples were stored at -20°C.
