# Supplementary material for: Identification and characterization of locus-specific methylation patterns within novel loci undergoing hypermethylation during breast cancer pathogenesis
Source: Breast Cancer Res. 2014 Feb 3;16(1):R17. doi: 10.1186/bcr3612 (PMC3978461; doi:10.1186/bcr3612)
Supplement: Additional file 3 — Details of the methylation-sensitive high-resolution melting assays used in the validation experiments. [file bcr3612-S3.doc]

Please notice that the names of the assays refer to closed functional element as mapped by NimbleScan software (e.g. mRNA or gene locus)

Legend:

Primer binding sites – in red. (Primers are included in length of the amplicons).

Original sequence was translated to bisulfite modified sequence using:

<http://www.urogene.org/methprimer/index1.html>

Fragment BC008699: chr14:37,123,572-37,123,689 (UCSC Genome Browser on Human Feb. 2009 (GRCh37/hg19) Assembly), Length: 118 bp

CCTGCCCAGTTCCCGGGAGGGCCAACCCCAGCCAGTAAGAAGACCTGAGGCGTAGGATCC

::||:::||||::++||||||::||::::||::||||||||||::|||||++||||||::

TTTGTTTAGTTTTCGGGAGGGTTAATTTTAGTTAGTAAGAAGATTTGAGGCGTAGGATTT

CTGCGCAGGAGGTTGCAGGAATGCCCCCGCTAGCCGCAAGGTTCCTGCTGGCCTGTAGAG

:||++:|||||||||:|||||||::::++:|||:++:||||||::||:|||::|||||||

TTGCGTAGGAGGTTGTAGGAATGTTTTCGTTAGTCGTAAGGTTTTTGTTGGTTTGTAGAG

CTTTCGTGATCCCCGCCAAGATGCGAACAGTAAGGTCCTCGTATGGATCGCAGTTTTTGT

:|||++||||:::++::||||||++||:||||||||::|++|||||||++:|||||||||

TTTTCGTGATTTTCGTTAAGATGCGAATAGTAAGGTTTTCGTATGGATCGTAGTTTTTGT

F: AGGATTTTTGCGTAGGAGGTTGT

R: ACGATCCATACGAAAACCTTACTA

Conditions:

Mg 3mM, 65 deg., 15,15,20 sec. pr. cycle

Fragment CA10: chr17:50,235,319-50,235,417 (UCSC Genome Browser on Human Feb. 2009 (GRCh37/hg19) Assembly), Length 99 bp

GCCGCTGGTGCGAAGAGAAGAGACACGCGAGCGGGGAGACCTCCAAGGCAGCGAGGCATC

+:++:|||||++|||||||||||:|++++||++||||||::|::||||:||++|||:||+

GTCGTTGGTGCGAAGAGAAGAGATACGCGAGCGGGGAGATTTTTAAGGTAGCGAGGTATC

GGACATGTGTCAGCACATCTGGGGCGCACATCCGTCGAGCCCGAGGGGAGATTTGCCGGA

+||:||||||:||:|:||:|||||++:|:||:++|++||::++||||||||||||:++||

GGATATGTGTTAGTATATTTGGGGCGTATATTCGTCGAGTTCGAGGGGAGATTTGTCGGA

ACAATTCAAACTGCGATATTGATCTTGGGGGTGACTGTCCCTGGCCGGCTGTCGGGTGGG

|:||||:|||:||++||||||||:||||||||||:|||:::|||:++|:|||++||||||

ATAATTTAAATTGCGATATTGATTTTGGGGGTGATTGTTTTTGGTCGGTTGTCGGGTGGG

F: GAGCGGGGAGATTTTTAAGGT

R: AAATTATTCCGACAAATCTCCCCT

Conditions:

Mg 3mM, 63 deg., 10,10,15 sec. pr. cycle

Fragment FLJ3247 (AK057009): chr2:223,162,979-223,163,068 (UCSC Genome Browser on Human Feb. 2009 (GRCh37/hg19) Assembly), Length 90 bp

CGCTCAGAAGCCGGTTCACCTCCTTCTCCACCGCGGCATTTCCAAAACAACAGGGACAAG

++:|:|||||:++|||:|::|::||:|::|:++++|:||||::||||:||:|||||:|||

CGTTTAGAAGTCGGTTTATTTTTTTTTTTATCGCGGTATTTTTAAAATAATAGGGATAAG

TCTCCCCGGCTCGCCGCAGGCCTGACCGCCCAGCTCCGCCAGGATTTGCAGAGAGCAGCG

|:|:::++|:|++:++:|||::|||:++:::||:|:++::||||||||:||||||:||++

TTTTTTCGGTTCGTCGTAGGTTTGATCGTTTAGTTTCGTTAGGATTTGTAGAGAGTAGCG

CGCTCCATTTGCAGAAAGGAAATCGAGTAGGTCCTCGCCCCCGACTGGTGCTTCTTGGGG

++:|::|||||:|||||||||||++|||||||::|++::::++|:|||||:||:||||||

CGTTTTATTTGTAGAAAGGAAATCGAGTAGGTTTTCGTTTTCGATTGGTGTTTTTTGGGG

F: GCGGTATTTTTAAAATAATAGGGATAAG

R: CGCGCTACTCTCTACAAATCCTAA

Conditions:

Mg 3mM, 61 deg., 20,20,30 sec. pr. cycle

Fragment HMX2: chr10:124,902,806-124,902,920 (UCSC Genome Browser on Human Feb. 2009 (GRCh37/hg19) Assembly), Length 115 bp

AGAACAACTAGGCGGGATGTACTTTTGAGCCCTGCCGGGTGTCTCCGATCGGAGTCTGGG

||||:||:||||++|||||||:|||||||:::||:++|||||:|:++||++||||:||||

AGAATAATTAGGCGGGATGTATTTTTGAGTTTTGTCGGGTGTTTTCGATCGGAGTTTGGG

GTTGAGATTTGGGCTGCACTTGTCCCCGGTGTGTCTCTCCGGCGGAGTACCCTGAAGGTG

|||||||||||||:||:|:||||:::++||||||:|:|:++|++|||||:::||||||||

GTTGAGATTTGGGTTGTATTTGTTTTCGGTGTGTTTTTTCGGCGGAGTATTTTGAAGGTG

CACGAGGTGGGGAGCATAGGCTGAGGTGGGTAATCGGGTCCTGGATAGAAACACAACCCT

:|++||||||||||:|||||:|||||||||||||++|||::||||||||||:|:||:::|

TACGAGGTGGGGAGTATAGGTTGAGGTGGGTAATCGGGTTTTGGATAGAAATATAATTTT

F: GCGGGATGTATTTTTGAGTTTTGT

R: CTCGTACACCTTCAAAATACTCC

Conditions:

Mg 3mM, 66 deg., 15,15,20 sec. pr. cycle

Fragment LHX1: chr17:35,297,992-35,298,091 (UCSC Genome Browser on Human Feb. 2009 (GRCh37/hg19) Assembly), Length 100 bp

AGCGCCAACGTGTCGGACAAGGAAGCGGGTAGCAACGAGAATGACGACCAGAACCTGGGC

||++::||++|||++||:|||||||++|||||:||++|||||||++|::||||::||||+

AGCGTTAACGTGTCGGATAAGGAAGCGGGTAGTAACGAGAATGACGATTAGAATTTGGGC

GCCAAGCGGCGGGGACCGCGCACCACCATCAAAGCCAAGCAGCTGGAGACGCTGAAGGCC

+::|||++|++||||:++++:|::|::||:||||::|||:||:||||||++:||||||:+

GTTAAGCGGCGGGGATCGCGTATTATTATTAAAGTTAAGTAGTTGGAGACGTTGAAGGTC

F: GTCGGATAAGGAAGCGGGTAGT

R: CGTCTCCAACTACTTAACTTTAATAATAATA

Conditions:

Mg 3mM, 63 deg., 10,10,15 sec. pr. cycle

Fragment NR2E1: chr6:108,485,970-108,486,088 (UCSC Genome Browser on Human Feb. 2009 (GRCh37/hg19) Assembly), Length 119 bp

GGCGCCCCACTAAGGAGGACACAGGCTCTGGTGTGTGTGGTGTGCGAGACCCCGAGCTCG

+|++::::|:|||||||||:|:|||:|:||||||||||||||||++|||:::++||:|++

GGCGTTTTATTAAGGAGGATATAGGTTTTGGTGTGTGTGGTGTGCGAGATTTCGAGTTCG

AGGCCGAGCCAAGGCTGGGCAGAAAGTTGCAATCACGTGCTGTCGGAGCCCACTGGAGCG

|||:++||::||||:||||:|||||||||:|||:|++||:|||++|||:::|:|||||++

AGGTCGAGTTAAGGTTGGGTAGAAAGTTGTAATTACGTGTTGTCGGAGTTTATTGGAGCG

CACAGCCCGCTCCCCCTGGGACGCCCAGGCGGAGGACCTGCTGCGCCCTCCCAGGGCTCG

:|:||::++:|:::::|||||++:::|||++|||||::||:||++:::|:::||||:|++

TATAGTTCGTTTTTTTTGGGACGTTTAGGCGGAGGATTTGTTGCGTTTTTTTAGGGTTCG

F: CGAGGTCGAGTTAAGGTTGGGT

R: ACCCTAAAAAAACGCAACAAATCCTC

Conditions:

Mg 3mM, 65 deg., 10,10,15 sec. pr. cycle

Fragment PHOX2B: chr4:41,753,256-41,753,361 (UCSC Genome Browser on Human Feb. 2009 (GRCh37/hg19) Assembly), Length 106 bp

CCTTACTGCACCTGGGGTGTGTCTCCGCGTGGTGCAGAGCGCGCGCTCTACTCCGGAAGC

::|||:||:|::||||||||||:|:++++|||||:||||++++++:|:||:|:++||||:

TTTTATTGTATTTGGGGTGTGTTTTCGCGTGGTGTAGAGCGCGCGTTTTATTTCGGAAGT

TACGGCCGGGTGCCGCGCCACCGCTGTGCGCCCTGGGCCTGATCCCTACGCCCTAGTCGA

||++|:++||||:++++::|:++:||||++:::||||::||||:::||++:::||||++|

TACGGTCGGGTGTCGCGTTATCGTTGTGCGTTTTGGGTTTGATTTTTACGTTTTAGTCGA

GTGCAGGGCAGGGCAATTTCGCCGTGGGTCCT

|||:||||:||||:|||||++:++|||||::|

GTGTAGGGTAGGGTAATTTCGTCGTGGGTTTT

F: GGGTGTGTTTTCGCGTGGTGT

R: TCGACTAAAACGTAAAAATCAAACCCAAAA

Conditions:

Mg 3mM, 61 deg., 10,10,15 sec. pr. cycle

Fragment SIX6: chr14:60,973,980-60,974,117 (UCSC Genome Browser on Human Feb. 2009 (GRCh37/hg19) Assembly), Length 138 bp

CCTGGCCAGAAGCTCCGGGATCGCAGCCCTCCCGGGTCCGGCTTCATCCCTGCCCGGCCA

::|||::|||||:|:++||||++:||:::|::++|||:++|:||:||:::||::++|::|

TTTGGTTAGAAGTTTCGGGATCGTAGTTTTTTCGGGTTCGGTTTTATTTTTGTTCGGTTA

CCGAGGCCCTCTTTTTCTGCACCGCGGATTCTCCTCCGCCTGCGTGTTCGGGGCCCTTGT

:++|||:::|:|||||:||:|:++++||||:|::|:++::||++||||++|||:::||||

TCGAGGTTTTTTTTTTTTGTATCGCGGATTTTTTTTCGTTTGCGTGTTCGGGGTTTTTGT

ATCCGATGTTTCTTTCTAAAAGTTGTCCTTCCGGCTGATTCGGAAGTCGCTCCAAGGGAA

||:++||||||:|||:||||||||||::||:++|:|||||++|||||++:|::|||||||

ATTCGATGTTTTTTTTTAAAAGTTGTTTTTTCGGTTGATTCGGAAGTCGTTTTAAGGGAA

F: GTTTTTTCGGGTTCGGTTTTATTTTTGT

R: CCGAATCAACCGAAAAAACAACTTTTAA

Conditions:

Mg 3mM, 59 deg., 15,15,20 sec. pr. cycle

Fragment WT1: chr11:32,456,867-32,456,962 (UCSC Genome Browser on Human Feb. 2009 (GRCh37/hg19) Assembly), Length 96 bp

CCGGCTCCGGGACACACGTGGAAGCCGGGTCCTGCAGCAAGAGGAAGTCCAGGATCGCGG

:++|:|:++|||:|:|++||||||:++|||::||:||:||||||||||::|||||++++|

TCGGTTTCGGGATATACGTGGAAGTCGGGTTTTGTAGTAAGAGGAAGTTTAGGATCGCGG

CGAGGAGACGGCGGGGCCCGGGCGCCTGGGCTGCCGTCCCGGCTCTGGGTGGGTGGGTGG

++||||||++|++|||::++||++::||||:||:++|::++|:|:|||||||||||||||

CGAGGAGACGGCGGGGTTCGGGCGTTTGGGTTGTCGTTTCGGTTTTGGGTGGGTGGGTGG

F: TATACGTGGAAGTCGGGTTTTGTA

R: CCAAAACCGAAACGACAACCCAAA

Conditions:

Mg 3mM, 58 deg., 10,10,20 sec. pr. cycle

Fragment TITF: chr14:36,992,328-36,992,413 (UCSC Genome Browser on Human Feb. 2009 (GRCh37/hg19) Assembly), Length 86 bp

CGCTGGCCCCTCGCGGAGCTTTCCCTGGCGCGACCTCACACGGTCGCTGCCTCTATTCCG

++:|||::::|++++|||:|||:::|||++++|::|:|:|++||++:||::|:||||:++

CGTTGGTTTTTCGCGGAGTTTTTTTTGGCGCGATTTTATACGGTCGTTGTTTTTATTTCG

ACCACGCTCTGCTTCGCTGGCTGCGGCTCCGCCAGGAATCCGAGGGGGCGCAGGCCCAGG

|::|++:|:||:||++:|||:||++|:|:++::||||||:++||||||++:|||:::|||

ATTACGTTTTGTTTCGTTGGTTGCGGTTTCGTTAGGAATTCGAGGGGGCGTAGGTTTAGG

F: GGAGTTTTTTTTGGCGCGATTTTATA

R: AATTCCTAACGAAACCGCAACCAA

Conditions:

Mg 3mM, 60 deg., 20,20,30 sec. pr. cycle

Fragment CRH: chr8:67,090,430-67,090,484 (UCSC Genome Browser on Human Feb. 2009 (GRCh37/hg19) Assembly), Length 83 bp

GCATACACACGTACACAGGCAGGGGCAGCCGGCTCCGCGGCGCACATCGCGGCAGCTCAG

|:|||:|:|++||:|:|||:|||||:||:++|:|:++++|++:|:||++++|:||:|:||

GTATATATACGTATATAGGTAGGGGTAGTCGGTTTCGCGGCGTATATCGCGGTAGTTTAG

GCAACGCAAAGTTGGTGGCGTGTTCCGTCCAGGCGCTCCCTACCTTCCCAGGCGCTTCGC

|:||++:|||||||||||++||||:++|::|||++:|:::||::||:::|||++:||++:

GTAACGTAAAGTTGGTGGCGTGTTTCGTTTAGGCGTTTTTTATTTTTTTAGGCGTTTCGT

F: TATACGTATATAGGTAGGGGTAGT

R: ACGAAACACGCCACCAACTTTA

Conditions:

Mg 3mM, 63 deg., 10,10,15 sec. pr. cycle

Fragment GHSR: chr3:172,167,580-172,167,683 (UCSC Genome Browser on Human Feb. 2009 (GRCh37/hg19) Assembly), Length 104 bp

GGACGCGGTCTGTGCGTCTCCTGCTCAGAGCCAGAAATCAGCACCCGAAGGCATGAGACT

|||++++||:||||++|:|::||:|:||||::||||||:||:|::++||||:||||||:|

GGACGCGGTTTGTGCGTTTTTTGTTTAGAGTTAGAAATTAGTATTCGAAGGTATGAGATT

GCCAGTTGCCAGCGAATTCACAAATCCGACCGGCCCCTCCCGGCCCACCGACCTCGGGAC

|::|||||::||++||||:|:||||:++|:++|::::|::++|:::|:++|::|++|||:

GTTAGTTGTTAGCGAATTTATAAATTCGATCGGTTTTTTTCGGTTTATCGATTTCGGGAT

CGCCCCAGGAACATATTCAGCACTGTGGCCAGCGCCACATCCATCCTACCGCAAAGCGCC

++::::|||||:|||||:||:|:|||||::||++::|:||::||::||:++:||||++:+

CGTTTTAGGAATATATTTAGTATTGTGGTTAGCGTTATATTTATTTTATCGTAAAGCGTC

F: GATTGTTAGTTGTTAGCGAATTTATAAATT

R: ATATAACGCTAACCACAATACTAAATATA

Conditions:

Mg 3mM, 60 deg., 20,20,30 sec. pr. cycle

Fragment HOX B13: chr17:46,810,857-46,810,932 (UCSC Genome Browser on Human Feb. 2009 (GRCh37/hg19) Assembly), Length 76 bp

GCAGCGCGACGCTCCCCTCTCCCGAAAGGTTGGCTCCACGGTCCCGCCGGCCGCGCAGGT

|:||++++|++:|::::|:|::++|||||||||:|::|++||::++:++|:++++:||||

GTAGCGCGACGTTTTTTTTTTTCGAAAGGTTGGTTTTACGGTTTCGTCGGTCGCGTAGGT

CTGGCTGAACTGCTTGGGGTCGCCCGGCTCCTCTCG

:|||:||||:||:|||||||++::++|:|::|:|++

TTGGTTGAATTGTTTGGGGTCGTTCGGTTTTTTTCG

F: GACGTTTTTTTTTTTCGAAAGGTTGGTTTT

R: ACGACCCCAAACAATTCAACCAAAC

Conditions:

Mg 3mM, 61 deg., 10,10,15 sec. pr. cycle

Fragment HTR1B: chr6:78,173,811-78,173,908 (UCSC Genome Browser on Human Feb. 2009 (GRCh37/hg19) Assembly), Length 98 bp

GACGGAGCCATAAAAGGGGGGACACGGGGGCTGGAGTTGCGGCTGCTCGGGCCGCGCCGC

||++|||::|||||||||||||:|++||||:||||||||++|:||:|++||:++++:++:

GACGGAGTTATAAAAGGGGGGATACGGGGGTTGGAGTTGCGGTTGTTCGGGTCGCGTCGT

CGCCACCGCCACCCTGGTCCCACGGGAGCCACTCGGAGCCATGCCACTGGGTGCGCGGGT

++::|:++::|:::||||:::|++||||::|:|++|||::|||::|:||||||++++|||

CGTTATCGTTATTTTGGTTTTACGGGAGTTATTCGGAGTTATGTTATTGGGTGCGCGGGT

F: GGATACGGGGGTTGGAGTTG

R: CGCGCACCCAATAACATAACT

Conditions:

Mg 3mM, 61 deg., 15,15,20 sec. pr. cycle

Fragment NKX2-3: chr10:101,293,836-101,293,948 (UCSC Genome Browser on Human Feb. 2009 (GRCh37/hg19) Assembly), Length 113 bp

GTCCTTGAACCCGTGGCACTCGGTAGAGAGAGAGGAGATGATCGGAAAGTGCGTGGGAAC

+|::|||||::++|||:|:|++||||||||||||||||||||++|||||||++||||||:

GTTTTTGAATTCGTGGTATTCGGTAGAGAGAGAGGAGATGATCGGAAAGTGCGTGGGAAT

AATGTCTATTCCGCGCGACCATAGCTCTCACATCCCTAAGGCGCCAGCCTTTTTTGAAAA

|||||:||||:++++++|::||||:|:|:|:||:::|||||++::||::|||||||||||

AATGTTTATTTCGCGCGATTATAGTTTTTATATTTTTAAGGCGTTAGTTTTTTTTGAAAA

TCCGTAACGTTTTGCTTTGTGTCCCAGGCTGCGGGCCTAATAGAAAACGCGCCGAACTTG

|:++|||++|||||:|||||||:::|||:||++||::||||||||||++++:++||:|||

TTCGTAACGTTTTGTTTTGTGTTTTAGGTTGCGGGTTTAATAGAAAACGCGTCGAATTTG

F: AAAGTGCGTGGGAATAATGTTTATTT

R: AAACCCGCAACCTAAAACACAAAA

Conditions:

Mg 3mM, 60 deg., 15,15,20 sec. pr. cycle

Fragment ONECUT: chr18:55,103,594-55,103,702 (UCSC Genome Browser on Human Feb. 2009 (GRCh37/hg19) Assembly), Length 109 bp

CATGAACAACCTCTACAGTCCCTACAAGGAGATGCCCGGCATGAGCCAGAGCCTGTCCCC

:|||||:||::|:||:|||:::||:|||||||||::++|:|||||::||||::|||:::+

TATGAATAATTTTTATAGTTTTTATAAGGAGATGTTCGGTATGAGTTAGAGTTTGTTTTC

GCTGGCCGCCACGCCGCTGGGCAACGGGCTAGGCGGCCTCCACAACGCGCAGCAGAGTCT

+:|||:++::|++:++:||||:||++||:||||++|::|::|:||++++:||:|||||:|

GTTGGTCGTTACGTCGTTGGGTAACGGGTTAGGCGGTTTTTATAACGCGTAGTAGAGTTT

GCCCAACTACGGTCCGCCGGGCCACGACAAAATGCTCAGCCCCAACTTCGACGCGCACCA

|:::||:||++||:++:++||::|++|:||||||:|:||::::||:||++|++++:|::|

GTTTAATTACGGTTCGTCGGGTTACGATAAAATGTTTAGTTTTAATTTCGACGCGTATTA

F: GAGATGTTCGGTATGAGTTAGAGTT

R: ACGAACCGTAATTAAACAAACTCTACTA

Conditions:

Mg 3mM, 58 deg., 20,20,30 sec. pr. Cycle

Fragment POU4F2: chr4:147,561,490-147,561,548 (UCSC Genome Browser on Human Feb. 2009 (GRCh37/hg19) Assembly), Length 100 bp

AGCATGGCCCACGCGCACGGGCTGCCGTCGCACATGGGCTGCATGAGCGACGTGGACGCC

||:||||:::|++++:|++||:||:++|++:|:|||||:||:|||||++|++||||++:+

AGTATGGTTTACGCGTACGGGTTGTCGTCGTATATGGGTTGTATGAGCGACGTGGACGTC

GACCCGCGGGACCTGGAGGCATTCGCCGAGCGCTTCAAGCAGCGACGCATCAAGCTGGGG

+|::++++|||::||||||:|||++:++||++:||:|||:||++|++:||:|||:|||||

GATTCGCGGGATTTGGAGGTATTCGTCGAGCGTTTTAAGTAGCGACGTATTAAGTTGGGG

F: GGTTGTCGTCGTATATGGGTTGT

R: CCCAACTTAATACGTCGCTACTTAAAA

Conditions:

Mg 3mM, 58 deg., 5,5,10 sec. pr. cycle

Fragment SLC38A4: chr12:47,224,928-47,225,029 (UCSC Genome Browser on Human Feb. 2009 (GRCh37/hg19) Assembly), Length 106 bp

ATTCGCCGTTTTCCCCCACAACCGGCACCTGCCCTGGCCCAGAGCGCAGCGTCCACCTGT

|||++:++||||:::::|:||:++|:|::||:::|||:::||||++:||++|::|::|||

ATTCGTCGTTTTTTTTTATAATCGGTATTTGTTTTGGTTTAGAGCGTAGCGTTTATTTGT

ACCACCGCTAGATGAAGAGTACCTCACCGCGCGCTGCCTGGGCGCACAGCTGGTGTGCCG

|::|:++:|||||||||||||::|:|:++++++:||::||||++:|:||:|||||||:++

ATTATCGTTAGATGAAGAGTATTTTATCGCGCGTTGTTTGGGCGTATAGTTGGTGTGTCG

ACCACCGCTAGATGAAGAGTACCTCACCGCGCGCTGCCTGGGCGCACAGCTGGTGTGCCG

++:|++::|::|:|::||||:|:|||||||:|::++|:|||++:||:|++|||:||::++

CGTTCGTTATTTTTTTTGGATTTAAGGGTGTTTTCGGTTTACGTTTTTCGGGGTTTTTCG

F: TAATCGGTATTTGTTTTGGTTTAGAG

R: AACGCGACACACCAACTATAC

Conditions:

Mg 3mM, 64 deg., 10,10,15 sec. pr. cycle

Fragment TMEM132D: chr12:130,387,867-130,387,972(UCSC Genome Browser on Human Feb. 2009 (GRCh37/hg19) Assembly), Length 106 bp

TGGTGCCACAGCGTCCCCATCTCAGACGGGCACATCCTGGAGACCCGGAGCGCAGATCCT

|||||::|:||++|::::||:|:|||++||:|:||::||||||::++|||++:||||::|

TGGTGTTATAGCGTTTTTATTTTAGACGGGTATATTTTGGAGATTCGGAGCGTAGATTTT

CCGCTCCCCGGCGCCGTCCAGGCGAACAAGAGACCGTCTCAGTCCCCTAGAGGCCCGCAG

:++:|:::++|++:++|::|||++||:||||||:++|:|:|||::::||||||::++:||

TCGTTTTTCGGCGTCGTTTAGGCGAATAAGAGATCGTTTTAGTTTTTTAGAGGTTCGTAG

CGGGGCCGGTGGCGAGGGAGCGCCCGGCTAGGGGCCCGAGCAGCCCGGGCGCCCTGCTCC

++|||:++||||++||||||++::++|:||||||::++||:||::++||++:::||:|::

CGGGGTCGGTGGCGAGGGAGCGTTCGGTTAGGGGTTCGAGTAGTTCGGGCGTTTTGTTTT

F: TATTTTAGACGGGTATATTTTGGAGATT

R: CCGCTACGAACCTCTAAAAAACTAAA

Conditions:

Mg 3mM, 60 deg., 10,10,15 sec. pr. cycle
